# Bullous pityriasis lichenoides et varioliformis acuta successfully treated with upadacitinib

**DOI:** 10.1016/j.jdcr.2025.06.051

**Published:** 2025-07-31

**Authors:** Jessica Houpe, Rebecca Gibons, Gia-Binh Nguyen, Vidya Medepalli, Marc Inglese

**Affiliations:** a Department of Dermatology, University of Central Florida/HCA Healthcare Dermatology Residency Program, Tallahassee, Florida; b Florida State University College of Medicine, Tallahassee, Florida; c University of Central Florida College of Medicine, Orlando, Florida

**Keywords:** papulosquamous, pityriasis lichenoides, skin of color, upadacitinib

## Introduction

Pityriasis lichenoides et varioliformis acuta (PLEVA) is a rare cutaneous inflammatory disorder that clinically appears as macules, papules, vesicles, pustules, and/or ulcers in various stages of healing.^1^ Bullae are rarely seen.^2^ The pathogenesis of PLEVA is unknown, but it is thought to be either related to an underlying infectious etiology or an inflammatory response to a T-cell dyscrasia.^1^

Historically, several treatment modalities have been used to treat PLEVA with various success rates such as oral antibiotics, topical corticosteroids, ultraviolet light therapy, and immunomodulating agents.^1^ Upadacitinib, an oral Janus kinase 1 inhibitor, has not been described in the literature as a treatment for PLEVA. We present a case of bullous PLEVA successfully treated with upadacitinib in an African American patient.

## Case report

**Fig 1:**
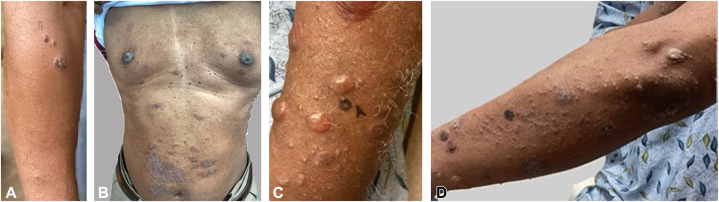
Various clinical presentations of the patient’s rash including eczematous, psoriasiform, and bullous. **A,** A few plaques with overlying scale on the right proximal arm. Clinically appeared as impetiginization of underlying atopic dermatitis. **B,** Psoriasiform-like plaques on the trunk. **C** and **D,** Papules and plaques with overlying scale, as well as tense bullae, some with hemorrhage distributed throughout his body. Clinically appeared as bullous dermatitis.

**Fig 2:**
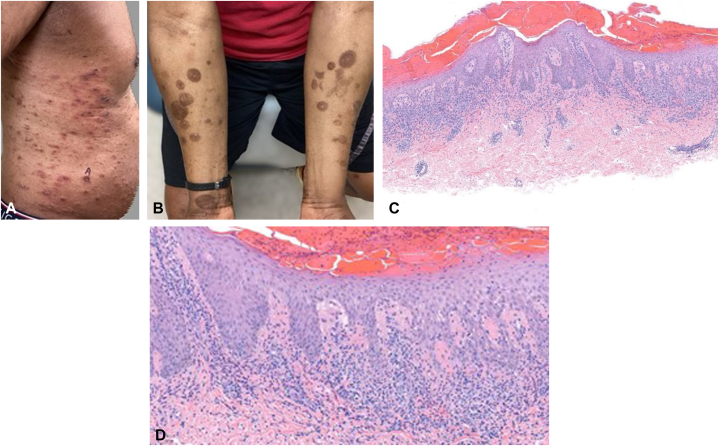
Pityriasiform appearance of rash with histology and posttreatment outcome. **A,** Eruption of papules, plaques, blisters in multiple stages of evolutions with a pityriasis-like appearance. **B,** Postinflammatory hyperpigmentation after 1 month treatment with upadacitinib. **C,** Low power histology showing wedge-shaped lichenoid infiltrate, superficial perivascular inflammation, overlying confluent parakeratosis, and scale. **D,** High power showing lymphocyte exocytosis with halo formation, spongiosis, basal vacuolar change, and extravasated erythrocytes.

A 67-year-old African American male with a past medical history of epilepsy on carbamazepine and levetiracetam presented with a 1-month history of a pruritic rash with overlying scale. His rash progressed over the next 6 months through multiple forms, including eczematous and psoriasiform. Each stage was biopsied and showed spongiosis, psoriasiform epidermal hyperplasia, and a superficial perivascular inflammation suggestive of chronic eczematous dermatitis. The patient later developed bullous lesions, which were rebiopsied, showing similar findings as before with negative direct immunofluorescence (Fig 1). His laboratory workup was negative for rapid plasma reagin, autoimmune blistering serologies, and hepatitis. During this time, the patient failed topical corticosteroids and topical ruxolitinib cream and worsened with oral deucravacitinib 6 mg daily and dupilumab 300 mg subcutaneous injections every 2 weeks. When the patient returned, his rash appeared pityriasiform and he underwent another biopsy which showed a lymphocytic infiltrate in the superficial vascular plexus, extending in a wedge-shaped pattern to the lower dermis with a lichenoid infiltrate, parakeratosis, and scattered extravasated erythrocytes, diagnostic for PLEVA (Fig 2). Given the clinical appearance of bullae but absence of blistering serologies, he was diagnosed with bullous PLEVA and treated with upadacitinib 15 mg daily. Treatment resulted in resolution of his rash with postinflammatory hyperpigmentation. He later suffered a breakthrough seizure from his underlying epilepsy which required prolonged hospitalization and discontinuation of upadacitinib. Based on current literature, the seizure was attributed to his underlying epilepsy, as there is no reported increased risk of seizures due to upadacitinib.

## Discussion

We describe a case of bullous PLEVA successfully treated with upadacitinib in an African American patient. Throughout the clinical course, the patient’s lesions progressed through various forms and stages (eg, papulo-squamous, bullous, eczematous). PLEVA entered the differential diagnosis after noting the polymorphic nature of the eruption and various stages of development of the lesions, features characteristic of PLEVA.^1^ Furthermore, the clinical, pathological, and negative autoimmune bullous serologies suggested a diagnosis of bullous PLEVA. Bullous formation is a rare skin manifestation of PLEVA. There are 2 distinct clinical entities that present with bullae—bullous PLEVA and PLEVA pemphigoides. In PLEVA pemphigoides, there is an overlap of PLEVA and bullous pemphigoid as these cases present with positive antibodies to bullous pemphigoid antigens.^2^ However, bullous formation with negative bullous pemphigoid antibodies may suggest bullous PLEVA. Similarly, bullous lichen planus (LP) is a rare manifestation of LP with negative bullous pemphigoid antibodies.^3^ Bullous LP appears clinically as LP lesions with the development of bullae or vesicles on or around the lesions.^3^^,^^4^ Compared to bullous PLEVA, bullous LP has similar histologic findings as LP with the addition of intrabasal bullae or vesicles due to an extensive inflammatory infiltrate that disrupts the dermoepidermal junction.^4^ Our case had bullae with negative autoimmune bullous disease serologies and histology characteristic of PLEVA, diagnosing bullous PLEVA. Additionally, we describe the clinical features of PLEVA in a patient with skin of color, contributing new images of PLEVA in darker skin types to the literature.

The etiology of PLEVA remains unclear. Current theories support an infectious cause or an inflammatory reaction, a T helper (Th) 1 response, to a T-cell dyscrasia,a Th2 mediated.^1^ Since our patient improved with upadacitinib and worsened with deucravacitinib and dupilumab, our patient’s bullous PLEVA may be an inflammatory response to a T-cell dyscrasia, as this pathogenic theory is an interplay between both Th1 and Th2 cells. Upadacitinib is a Janus kinase 1 inhibitor that functions through the Janus kinase-signal transducers and activators of transcription pathway to prevent transcription of multiple inflammatory cytokines to suppress both Th1 and Th2 responses.^5^ Thus, upadacitinib is more likely to maintain a balance between Th1 and Th2 responses as it modulates the immune system.^6^ In contrast, deucravacitinib, a tyrosine kinase 2 inhibitor, selectively inhibits the Th1 response while dupilumab, an interleukin 4 alpha receptor inhibitor, selectively inhibits the Th2 response; both of which would result in an imbalance of the Th1 and Th2 cells and worsening of a T-cell dyscrasia.^7^^,^^8^

Given that our patient’s PLEVA worsened after treatment with both deucravacitinib and dupilumab, which target different cytokine pathways than upadacitinib, we can support the pathogenic theory of PLEVA as an inflammatory response to a T-cell dyscrasia by considering how these treatments differ. Upadacitinib may be a treatment option for patients who have failed the first-line treatments for PLEVA. Furthermore, this case highlights how the classic features of PLEVA can be more subtle and challenging to observe in patients with skin of color, making it an excellent example for practicing dermatologists.

## Conflicts of interest

None disclosed.
